# Engagement in the pre‐exposure prophylaxis (PrEP) cascade among a respondent‐driven sample of sexually active men who have sex with men and transgender women during early PrEP implementation in Zimbabwe

**DOI:** 10.1002/jia2.25873

**Published:** 2022-02-11

**Authors:** Lauren E. Parmley, Tiffany G. Harris, Innocent Chingombe, Munyaradzi Mapingure, Owen Mugurungi, John H. Rogers, Perpetua Gozhora, Yingfeng Wu, Chesterfield Samba, Godfrey Musuka, Avi J. Hakim

**Affiliations:** ^1^ ICAP at Columbia University New York New York USA; ^2^ Department of Epidemiology, Mailman School of Public Health Columbia University New York New York USA; ^3^ ICAP at Columbia University Harare Zimbabwe; ^4^ Zimbabwe Ministry of Health and Child Care Harare Zimbabwe; ^5^ Division of Global HIV & TB U.S. Centers for Disease Control Harare Zimbabwe; ^6^ GALZ Harare Zimbabwe; ^7^ Division of Global HIV & TB U.S. Centers for Disease Control Atlanta Georgia USA

**Keywords:** PrEP, men who have sex with men, transgender people, HIV prevention, Africa, key and vulnerable populations

## Abstract

**Introduction:**

Zimbabwe is scaling up pre‐exposure prophylaxis (PrEP) for key populations, including men who have sex with men (MSM) and transgender women (TGW). To assess implementation and inform HIV programming, we evaluated gaps in PrEP awareness, uptake and use, and correlates of awareness and uptake among a sample of MSM, TGW and genderqueer individuals (GQ) in Harare and Bulawayo, Zimbabwe.

**Methods:**

Respondent‐driven sampling was used to recruit 1194 MSM and 344 TGW/GQ aged ≥18 to participate in a cross‐sectional survey assessing HIV‐related outcomes in 2019. Consenting participants completed a questionnaire on socio‐demographic information, sexual risk practices and engagement in HIV services and underwent HIV testing. Descriptive statistics were used to assess the PrEP cascade. Multiple logistic regression models were used to identify factors associated with PrEP awareness and uptake among HIV‐negative participants. Data were unweighted as the sample did not reach convergence on key estimates.

**Results:**

Among the 1167 HIV‐negative participants, most (79.2%) were MSM compared to TGW/GQ (20.8%). Median age was 24 years. Overall, 45.8% were aware of PrEP and of those, 31.3% had ever taken PrEP. Most (71.1%) reporting never taking PrEP were willing to start PrEP; the main reasons for never starting PrEP included not knowing where to access it (24.8%) and fearing side effects (20.4%). Among those who had ever taken PrEP, 74.9% had taken PrEP in the last 6 months; of these, 42.4% had taken PrEP the day of or day preceding the survey. Side effects represented the most common (59.5%) reason for discontinuing PrEP. MSM (adjusted odds ratio [aOR]: 2.5, 95% confidence interval [CI]: 1.8–3.6) and TGW/GQ in Harare (aOR: 3.1, 95% CI: 2.1–4.7), and TGW/GQ in Bulawayo (aOR: 2.4, 95% CI: 1.1–5.3) had higher awareness of PrEP than MSM in Bulawayo. Overall, TGW/GQ were more likely to have ever taken PrEP compared to MSM (aOR: 1.6, 95% CI: 1.01–2.4).

**Conclusions:**

Findings emphasize the need for tailored interventions to promote PrEP among key populations. As HIV programs in Zimbabwe continue to expand PrEP services, these data, including barriers to starting and continuing PrEP, can inform strategies to address gaps along the PrEP cascade.

## 1 INTRODUCTION

Zimbabwe has made substantial progress towards reaching the UNAIDS 95‐95‐95 targets: 87% of adult people living with HIV (PLHIV) are aware of their HIV status; of these, 97% are receiving antiretroviral therapy (ART); and of these, 90% are virally suppressed [1]. New HIV infections have declined by 66% since 2010, and as of 2020, Zimbabwe's incidence to prevalence ratio was 2.0%, below the commonly used benchmark of 3.0% [2]. Strengthening HIV prevention efforts to ensure persons at highest risk of acquiring HIV, including key populations (KP), such as gay, bisexual and other cisgender men who have sex with men (MSM) and transgender women (TGW) or women assigned male at birth, could help Zimbabwe sustain these gains and achieve HIV epidemic control [3].

Globally, MSM and TGW have 22 and 12 times higher risk of HIV acquisition, respectively, than men in the general population and together, represent nearly 20% of all new HIV infections [3]. In Zimbabwe, where same‐sex sexual relations are illegal and highly stigmatized [4, 5], HIV prevalence among MSM, TGW and genderqueer/non‐binary individuals (GQ) assigned male at birth is more than two times that of the general adult male population in urban areas (Harare: 21.4% vs. 10.0%; Bulawayo: 23.4% vs. 13.8%) and recent HIV infection among them is 1.1% [6, 7].

Given this disproportionate burden, KPs have been targeted for pre‐exposure prophylaxis (PrEP) [8]. PrEP, which can reduce HIV acquisition risk by up to 99%, is being scaled‐up for populations at substantial HIV risk in sub‐Saharan Africa [9]. In Zimbabwe, a plan to scale‐up PrEP between 2018 and 2020 was developed, following PrEP's introduction in 2016 [8, 10]. Zimbabwe's targets to enrol individuals on PrEP under the U.S. President's Emergency Plan for AIDS Relief have dramatically increased from just 2769 in 2018 to 22,799 in 2021 [11].

Like the scale‐up of ART, successful PrEP delivery requires coordination and management across all levels of the health sector—from demand creation to supply [12]. Equally, PrEP program effectiveness requires several factors to be met (e.g. awareness, access, acceptability, uptake and adherence). HIV prevention cascades, modelled after the HIV treatment cascade, can assist in identifying gaps along the continuum of services, which shape the effective use of HIV prevention methods, such as PrEP, and provide a useful programmatic framework to target interventions [12, 13]. We assessed the PrEP cascade, including awareness, uptake and use, and correlates of awareness and uptake, among MSM and TGW/GQ during early PrEP implementation in Zimbabwe.

## 2 METHODS

### 2.1 Setting

The survey was conducted in Zimbabwe's two largest cities, Harare and Bulawayo, where HIV prevalence among the general population was 12.6% and 14.0%, respectively [1]. In both cities, selected public facilities, including those already offering ART and serving KP, offered PrEP. KP organizations, the primary implementors of PrEP services for KP, were operational in both cities; however, central offices and activities of these organizations were based in Harare. At the time of the survey, there were no MSM and/or TGW/GQ‐specific PrEP campaigns.

### 2.2 Data collection

From March to July 2019, MSM and TGW/GQ were recruited to participate in the cross‐sectional survey using respondent‐driven sampling (RDS) [14], a chain referral approach to reach populations for whom no sampling frame exists. Purposively selected “seeds,” well‐networked and respected MSM and TGW/GQ, were recruited into the survey via KP‐led organizations and community mobilizers and asked to recruit three of their peers, who were enrolled (if eligible and provided consent) and were asked to recruit three of their peers, with the aim of achieving a final sample independent of “seeds.” Seeds were recruited to ensure diversity in demographics, awareness of HIV status and engagement with KP‐friendly organizations. An electronic coupon manager was used to track recruiter–recruit relationships and coupon eligibility. Individuals were eligible if they were male assigned at birth; were aged ≥18 years; had engaged in anal or oral sex with a man in the past 12 months; and spoke English, Shona or Ndebele. Sample sizes of 718 participants in Harare and 820 participants in Bulawayo were needed to estimate HIV prevalence and viral load suppression, the primary aims of the survey, with precision at 95% confidence interval (CI) level. An initial 14 seeds (Harare: 8, Bulawayo: 6) were recruited, with five additional seeds recruited (Harare: 3, Bulawayo: 2) as accrual slowed.

All participants provided written informed consent for survey participation and biomarker testing separately. The questionnaire, adapted from the World Health Organization Biobehavioral Survey Guidelines [15], was administered via tablet in English, Shona or Ndebele at a private office. PrEP‐specific questions were restricted to participants who self‐reported an unknown or HIV‐negative status. After interview completion, participants underwent HIV testing using a three‐test algorithm. Participants were referred to their choice of KP‐friendly organization or health facility for PrEP or HIV care according to HIV test result. Participants were reimbursed US$5 to cover participation time and transportation and an additional US$5 for each recruit (maximum of three).

Several procedures were taken to protect participant privacy and confidentiality. Survey investigators worked closely with GALZ, an organization serving lesbian, gay, bisexual and transgender communities, throughout the survey to ensure safe and appropriate methods and implementation. Participants were provided the option of attending one of two survey sites in each city (a KP‐organization or non‐KP‐affiliated organization) all of which had private security. To ensure participant confidentiality, all staff underwent KP‐sensitivity training and signed confidentiality agreements. Ethical and administrative approvals were received from the Centers for Disease Control and Prevention (2018‐444), Columbia University Institutional Review Board (AAAR8950) and the Medical Research Council of Zimbabwe (MRCZ/A/2156). Additional information on the survey methods and results of the primary survey objectives have been published elsewhere [16].

### 2.3 Measures

Primary analyses were restricted to participants who self‐reported having a negative or unknown HIV status and who tested HIV negative during the survey. The PrEP cascade was conditional and included four steps: aware of PrEP, ever taken PrEP, taken PrEP in the last 6 months and currently on PrEP. Awareness of PrEP was assessed with the question “PrEP is a medicine that can prevent HIV. It is taken by HIV‐negative people. Have you heard of PrEP?,” with participants who answered “Yes” classified as aware. Ever taken PrEP and taken PrEP in the last 6 months were assessed with the questions “Have you ever taken PrEP?” and “In the last 6 months, have you taken PrEP?,” respectively. Current PrEP use was assessed with the question “When was the last time you took PrEP?” with participants who answered “Yesterday or today” classified as currently taking PrEP. Additional variables related to frequency of PrEP use, willingness to take PrEP and reasons for not taking or stopping PrEP were also assessed. Other measures analysed included the Alcohol Use Disorders Identification Test (AUDIT) [17], the Patient Health Questionnaire‐2 (PHQ2) [18] and a series of five HIV transmission questions used to assess comprehensive HIV knowledge, according to the UNAIDS definition [19]. A two‐step question was used to determine gender identity; participants were first asked their current sex or gender followed by their sex assigned at birth. Participants who identified as male were categorized as MSM. Participants who identified as female/trans female/trans women were categorized as TGW and those who identified as GQ, a non‐binary gender term used in Zimbabwe, were categorized as GQ. In analysis, TGW and GQ were combined due to small sample sizes and based on feedback from in‐country stakeholders. Network size was determined using a series of questions aligned to the eligibility criteria and referenced the number of eligible individuals who the participant had seen within the last 2 weeks.

### 2.4 Statistical analysis

Data were analysed in SAS 9.4 (Cary, NC) and recruitment diagnostics (e.g. recruitment tree, recruits by seed and wave, homophily, convergence and bottleneck plots) were explored using RDS‐Analyst 1.8 (Los Angeles, CA) [20]. The sample did not reach convergence on key estimates, including those related to primary study objectives, such as HIV prevalence. Due to lack of convergence and inability to meet RDS estimator conditions or assumptions, analyses were unweighted and did not account for sampling design. Bivariate analyses included chi‐square tests with continuity adjustment and Fisher's exact tests. Multiple logistic regression models with backward selection were used to identify factors associated with PrEP awareness and uptake adjusting for variables that were significantly (*p* < 0.05) associated with outcome variables in bivariate logistic regression. Models were conceptualized around demographic factors, social networks and sources of information, and factors associated with HIV risk (e.g. condomless receptive anal sex [CRAI], transactional sex, substance use and sexually transmitted infection [STI] history). An interaction term between city and KP was included in the first multivariable model due to evidence of interaction. Complete case analysis was used because <5% of data were missing. Tests for collinearity, including examination of the correlation matrix and investigation of the variance inflation factor and tolerance of the models, were conducted.

To identify gaps along the PrEP cascade for participants who may have most benefited from PrEP (those with new HIV diagnoses), a separate sub‐analysis among participants who self‐reported having a negative or unknown HIV status but tested HIV positive was conducted, and for the last two measures of the cascade, the denominator excluded participants with viral load suppression (<1000 copies/ml) as we assumed these participants were aware of their HIV infection and on treatment.

## 3 RESULTS

### 3.1 Recruitment statistics and participant characteristics

A total of 19 seeds participated (MSM: 12; TGW/GQ: 7). The mean number of recruits per seed and the longest recruitment wave were 64 and 17, respectively, in Harare and 102 and 14, respectively, in Bulawayo. Overall, 1927 coupons were distributed in Harare (return rate: 42.8%) and 1913 coupons were distributed in Bulawayo (return rate: 52.3%). In total, 1845 individuals were screened for eligibility (Harare: 836; Bulawayo: 1009). Of these, 1538 participants were recruited in both sites ([Harare: 718; Bulawayo: 820]; [MSM: 1194; TGW/GQ: 344]) and 89.5% (1377/1538) self‐reported an HIV negative or unknown status ([Harare: 92.1%; Bulawayo: 87.3%]; [MSM: 89.4%; TGW/GQ: 89.8%]). Of these, 84.7% (1167/1377) tested HIV negative (Table 1). Among participants who self‐reported an HIV negative or unknown status and tested HIV negative, the majority were MSM (79.2% [924/1167]) and aged 18–24 years (53.7% [627/1167]).

**Table 1:** Demographic characteristics among HIV‐negative men who have sex with men and transgender women/genderqueer individuals, Zimbabwe, 2019

	MSM (*n* = 924, 79.2%)		TGW/GQ (*n* = 243, 20.8%)		Total (*n* = 1167, 100%)	
	*n*	col%	*n*	col%	*n*	col%
City						
Harare	344	37.2	201	82.7	545	46.7
Bulawayo	580	62.8	42	17.3	622	53.3
Age (years)						
18–24	451	48.8	176	72.4	627	53.7
25–34	325	35.2	59	24.3	384	32.9
35 or older	55	10.1	101	16.2	156	13.37
Median (IQR)	25 (21–31)		22 (20–26)		24 (21–30)	
Race						
Black African	907	98.2	240	98.8	1147	98.3
Non‐Black African	17	1.8	3	1.2	20	1.7
Nationality						
Zimbabwean	915	99.0	239	98.4	1154	98.9
Other African	9	1.0	4	1.7	13	1.1
Employment status						
Self‐employed	215	23.3	47	19.3	262	22.5
Employed full‐time	118	12.8	29	11.9	147	12.6
Employed part‐time	99	10.7	23	9.5	122	10.5
Full‐time student	137	14.8	47	19.3	184	15.8
Retired	3	0.3	0	0	3	0.3
Unemployed	352	38.1	97	39.9	449	38.5
Highest education achieved						
Primary or less	48	5.2	8	3.3	56	4.8
Secondary	649	70.2	176	72.4	825	70.7
Tertiary	185	20.0	40	16.5	225	19.3
Vocational	42	4.6	19	7.8	61	5.2
Marital status						
Single, never married	760	82.3	230	94.7	990	84.8
Married/cohabitating	70	7.6	5	2.1	75	6.4
Separated/divorced	86	9.3	8	3.3	94	8.1
Widowed	8	0.9	0	0	8	0.7
Regular place to sleep at night						
Yes	910	98.5	241	99.2	1151	98.6
No	14	1.5	2	0.8	16	1.4
Sexual orientation^a^						
Gay/homosexual	467	50.6	184	75.7	651	55.8
Bisexual	456	49.6	59	24.3	515	44.2
Straight/heterosexual	0	0	0	0	0	0

Abbreviations: IQR, interquartile range; MSM, men who have sex with men; TGW/GQ, transgender women/genderqueer.

^a^ *n* = 1 don't know/refuse to answer.

### 3.2 PrEP cascade

**Figure 1:**
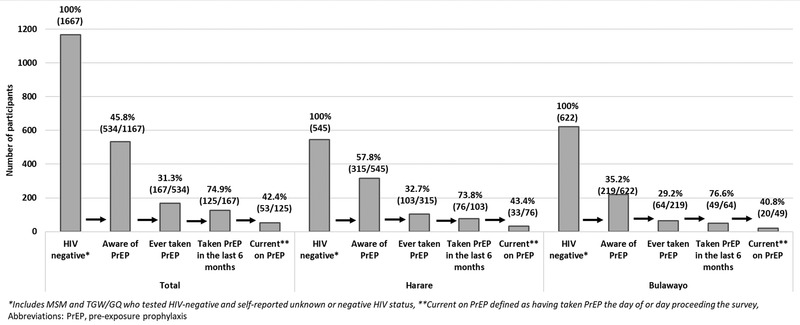
PrEP cascade among HIV‐negative men who have sex with men and transgender women/genderqueer individuals by city, Zimbabwe, 2019.

**Figure 2:**
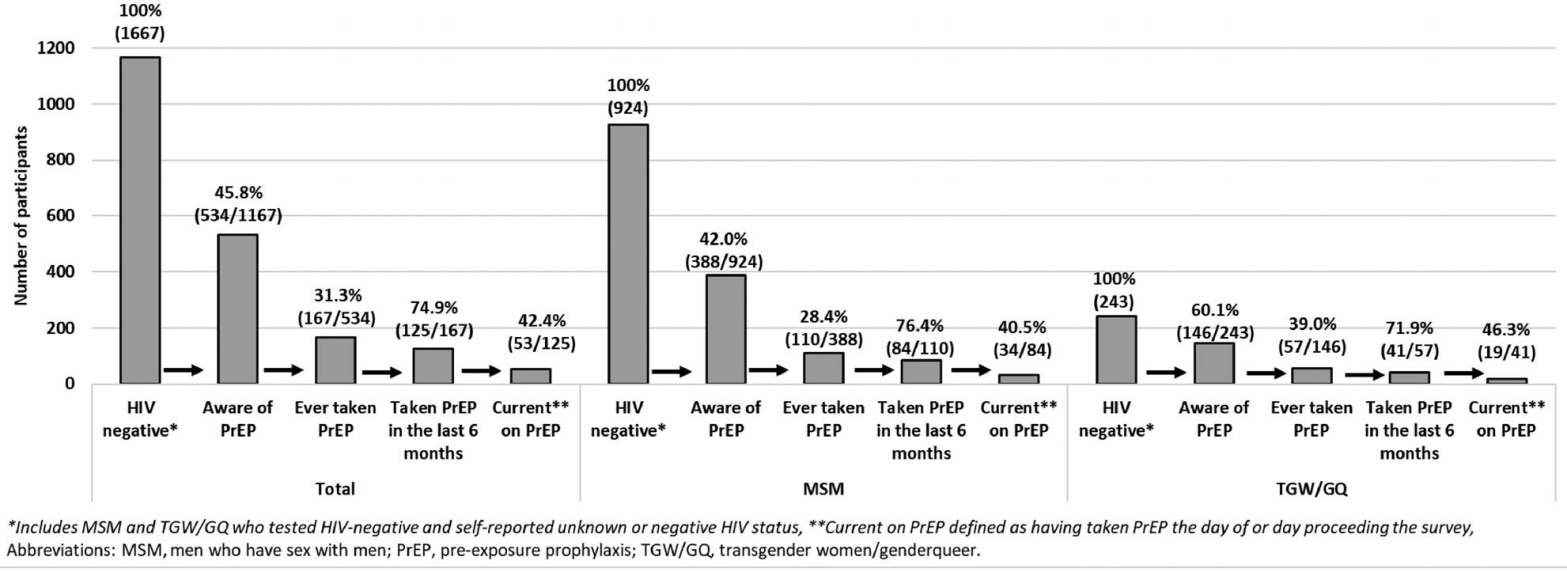
PrEP cascade among HIV‐negative men who have sex with men and transgender women/genderqueer individuals by key population, Zimbabwe, 2019.

**Figure 3:**
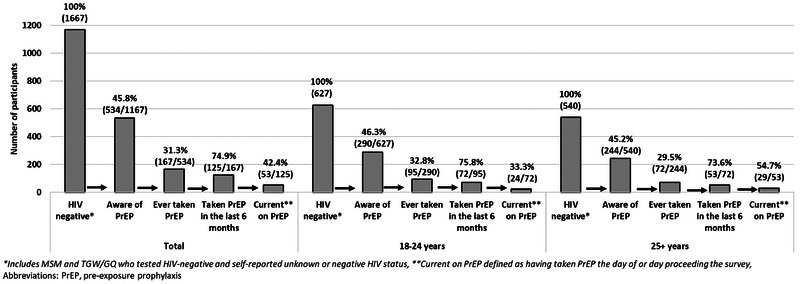
PrEP cascade among HIV‐negative men who have sex with men and transgender women/genderqueer individuals by age group, Zimbabwe, 2019.

PrEP cascades by city, KP and age are shown in Figures 1, 2, 3. Overall, 45.8% (534/1167) of participants were aware of PrEP. Of these, 31.3% (167/534) had ever taken PrEP. Most (71.1% [261/367]) who reported never taking PrEP were willing to start PrEP. The main reasons for never starting PrEP included not knowing where to access PrEP (24.8% [91/367]), fearing side effects (20.4% [75/367]), not feeling at risk for HIV (19.6% [72/367]), not wanting to start PrEP (13.6% [50/367]) and insufficient information about PrEP (6.0% [22/367]). Among those who had ever taken PrEP, 74.9% (125/167) had taken it in the last 6 months; reasons for discontinuing PrEP included side effects (59.5% [25/42]), trust in partner (7.1% [3/42]), inability to access PrEP (4.8% [2/42]), concern about others finding out (2.4% [1/42]) or other reasons (26.2% [11/42]). Most PrEP users in the last 6 months reported taking PrEP daily (70.4% [88/125]) and 42.4% (53/125) were currently taking PrEP.

### 3.3 Factors associated with PrEP awareness

In multiple logistic regression (Table 2), Harare MSM (adjusted odds ratio [aOR]: 2.5, 95% CI: 1.8–3.6), Harare TGW/GQ (aOR: 3.1, 95% CI: 2.1–4.7) and Bulawayo TGW/GQ (aOR: 2.4, 95% CI: 1.1–5.3) had higher awareness of PrEP than Bulawayo MSM. Participants were more likely to be aware of PrEP if they had attended secondary (aOR: 2.7, 95% CI: 1.04–6.8) or tertiary school (aOR: 8.5, 95% CI: 3.2–22.8) compared to primary school or less, had self‐reported a larger network than the sample median (aOR: 1.4, 95% CI: 1.03–1.8), had ever spoken with a peer educator/outreach worker (aOR: 1.8, 95% CI: 1.3–2.5), had ever tested for HIV (aOR: 2.3, 95% CI: 1.5–3.6), had received free condoms in the last 12 months (aOR: 1.4, 95% CI: 1.03–1.9), had received information on condom use and safe sex in the last 12 months (aOR: 2.0, 95% CI: 1.5–2.6), had been diagnosed with an STI in the last 12 months (aOR: 2.1, 95% CI: 1.3–3.4), answered all HIV transmission knowledge questions correctly (aOR: 1.4, 95% CI: 1.01–2.0) or had major depressive disorder (aOR: 2.2, 95% CI: 1.4–3.4). Participants were less likely to be aware of PrEP if they were aged ≥35 years (aOR: 0.5, 95% CI: 0.3–0.8) compared to those aged 18–24 years or had used non‐injection drugs recreationally in the past 6 months (aOR: 0.5, 95% CI: 0.4–0.6).

**Table 2:** Associations with PrEP awareness among HIV‐negative men who have sex with men and transgender women/genderqueer individuals, Zimbabwe, 2019

	Total (*n* = 1167)		Aware of PrEP (*n* = 534)		Unaware of PrEP (*n* = 633)		OR (95% CI)	*p*‐Value	aOR^g^ (95% CI)	*p*‐Value
	*n*	col%	*n*	row%	*n*	row%				
Key population, by city								**<0.0001**		**<0.0001**
Harare										
MSM	344	29.5	193	56.1	151	43.9	**2.5 (1.9–3.3)**		**2.5 (1.8–3.6)**	
TGW/GQ	201	17.2	122	60.7	79	39.3	**3.1 (2.2–4.3)**		**3.1 (2.1–4.7)**	
Bulawayo										
MSM	580	49.7	195	33.6	385	66.4	1		1	
TGW/GQ	42	3.6	24	57.1	18	42.9	**2.6 (1.4–5.0)**		**2.4 (1.1–5.3)**	
Age (years)								**<0.0001**		**0.0031**
18–24	627	53.7	290	46.3	337	53.7	1		1	
25–34	384	32.9	197	51.3	187	48.7	1.2 (0.9–1.6)		1.2 (0.9–1.6)	
35 or older	156	13.4	47	30.1	109	69.9	**0.5 (0.3–0.7)**		**0.5 (0.3–0.8)**	
Highest education attended								**<0.0001**		**<0.0001**
Primary or less	56	4.8	6	10.7	50	89.3	1		1	
Secondary	825	70.7	339	41.1	486	58.9	**5.8 (2.5–13.7)**		**2.7 (1.04–6.8)**	
Tertiary	225	19.3	163	72.4	62	27.6	**21.9 (8.9–53.7)**		**8.5 (3.2–22.8)**	
Vocational	61	5.2	26	42.6	35	57.4	**6.2 (2.3–16.6)**		2.0 (0.7–5.9)	
Network size								**0.0005**		**0.0315**
Median (4) or less	598	51.2	244	40.8	354	59.2	1		1	
Greater than the median	569	48.8	290	51.0	279	49.0	**1.5 (1.2–1.9)**		**1.4 (1.03–1.8)**	
Received free condoms in the last 12 months								**<0.0001**		**0.0336**
Yes	779	66.8	399	51.2	380	48.8	**2.0 (1.5–2.5)**		**1.4 (1.03–1.9)**	
No	388	33.2	135	34.8	253	65.2	1		1	
Received info on condom use and safe sex in the last 12 months								**<0.0001**		**<0.0001**
Yes	625	53.6	363	58.1	262	41.9	**3.0 (2.4–3.8)**		**2.0 (1.5–2.6)**	
No	542	46.4	171	31.5	371	68.5	1		1	
Ever spoken with a peer educator/outreach worker								**<0.0001**		**0.0002**
Yes	685	58.7	363	53.0	322	47.0	**2.1 (1.6–2.6)**		**1.8 (1.3–2.5)**	
No	482	41.3	171	35.5	311	64.5	1		1	
Ever tested for HIV								**<0.0001**		**0.0004**
Yes	990	84.8	494	49.9	496	50.1	**3.4 (2.4–5.0)**		**2.3 (1.5–3.6)**	
No	177	15.2	40	22.6	137	77.4	1		1	
Told by healthcare provider they had an STI in the last 12 months								**<0.0001**		**0.0025**
Yes	115	9.9	75	65.2	40	34.8	**2.4 (1.6–3.6)**		**2.1 (1.3–3.4)**	
No	1052	90.1	459	43.6	593	56.4	1		1	
Answered all HIV transmission knowledge questions correctly								**<0.0001**		**0.0412**
Yes	836	71.6	424	50.7	412	49.3	**2.1 (1.6–2.8)**		**1.4 (1.01–2.0)**	
No	331	28.4	110	33.2	221	66.8	1		1	
Think HIV messages they have seen apply to MSM/TGW^a^								0.0845		
Yes	887	76.1	418	47.1	469	52.9	1.3 (1.0–1.7)			
No	279	23.9	115	41.2	164	58.8	1			
Prefer to receive information about HIV from healthcare providers								0.0744		
Yes	910	78.0	429	47.1	481	52.9	1.3 (1.0–1.7)			
No	257	22.0	105	40.9	152	59.1	1			
Prefer to receive information about HIV from peer educators								**<0.0001**		
Yes	589	50.5	311	52.8	278	47.2	**1.8 (1.4–2.3)**			
No	578	49.5	223	38.6	355	61.4	1			
Participated in transactional sex in the last 6 months^b^								**0.0181**		
Yes	81	7.1	47	58.0	34	42.0	**1.7 (1.1–2.7)**			
No	1067	92.9	473	44.3	594	55.7	1			
Last sex with main male partner was CRAI^c^								**0.0124**		
Yes	166	15.0	90	54.2	76	45.8	**1.5 (1.1–2.1)**			
No	943	85.0	412	43.7	531	56.3	1			
Alcohol dependence^d^								**0.0422**		
Yes	245	21.0	98	40.0	147	60.0	**0.7 (0.6–0.99)**			
No	922	79.0	436	47.3	486	52.7	1			
Used non‐injection drugs in the past 6 months^e^								**<0.0001**		**<0.0001**
Yes	527	45.2	170	32.3	357	67.7	**0.4 (0.3–0.5)**		**0.5 (0.4–0.6)**	
No	640	54.8	364	56.9	276	43.1	1		1	
Major depressive disorder^f^								**<0.0001**		**0.0007**
Major depressive disorder likely	134	11.5	83	61.9	51	38.1	**2.1 (1.5–3.0)**		**2.2 (1.4–3.4)**	
Not depressed	1033	88.5	451	43.7	582	56.3	1		1	

Note: Statistically significant results are in boldface (*p* < 0.05).

Abbreviations: aOR, adjusted odds ratio; AUDIT, Alcohol Use Disorders Identification Test; CI, confidence interval; CRAI, condomless receptive anal intercourse; MSM, men who have sex with men; OR, odds ratio; PHQ‐2, Patient Health Questionnaire; PrEP, pre‐exposure prophylaxis STI, sexually transmitted infection; TGW/GQ, transgender women/genderqueer.

^a^ *n* = 1 missing.

^b^ *n* = 19 missing.

^c^ *n* = 58 missing.

^d^ AUDIT score ≥15.

^e^ Most reported non‐injection drugs used in the past 6 months included tobacco ([70.0%]; [369/527]) and marijuana ([65.8%]; [347/527]).

^f^ PHQ‐2 score ≥3.

^g^ Adjusted for all variables included in the final multiple logistic regression model.

### 3.4 Factors associated with PrEP uptake

Among those aware of PrEP, participants were more likely to have ever taken PrEP if they were TGW/GQ (aOR: 1.6, 95% CI: 1.01–2.4; Table 3), had a self‐reported network size greater than the sample median (aOR: 1.6, 95% CI: 1.1–2.4), had ever spoken with a peer educator/outreach worker (aOR: 1.6, 95% CI: 1.02–2.5), had received free condoms in the last 12 months (aOR: 1.8, 95% CI: 1.1–2.9), had been diagnosed with an STI in the last 12 months (aOR: 2.4, 95% CI: 1.4–4.1) or had participated in transactional sex in the last 6 months (aOR: 2.1, 95% CI: 1.1–4.1) and were less likely to have ever taken PrEP if they had used non‐injection drugs recreationally in the past 6 months (aOR: 0.5, 95% CI: 0.3–0.8).

**Table 3:** Associations with PrEP uptake among HIV‐negative men who have sex with men and transgender women/genderqueer individuals, Zimbabwe, 2019

	Total (*n* = 534)		Used PrEP (*n* = 167)		Not used PrEP (*n* = 367)		OR (95% CI)	*p*‐Value	aOR^f^ (95% CI)	*p*‐Value
	*n*	col%	*n*	row%	*n*	row%				
Key population								**0.0180**		**0.0453**
MSM	388	72.7	110	28.4	278	71.6	1		1	
TGW/GQ	146	27.3	57	39.0	89	61.0	**1.6 (1.1–2.4)**		**1.6 (1.01–2.4)**	
City								0.3945		
Bulawayo	219	41.0	64	29.2	155	70.8	1			
Harare	315	59.0	103	32.7	212	67.3	1.2 (0.8–1.7)			
Age (years)								0.6898		
18–24	290	54.3	95	32.8	195	67.2	1			
25–34	197	36.9	59	29.9	138	70.1	0.9 (0.6–1.3)			
35 or older	47	8.8	13	27.7	34	72.3	0.8 (0.4–1.6)			
Highest education attended								0.7507		
Primary or less	6	1.1	1	16.7	5	83.3	1			
Secondary	339	63.5	106	31.3	233	68.7	2.3 (0.3–19.7)			
Tertiary	163	30.5	50	30.7	113	69.3	2.2 (0.3–19.4)			
Vocational	26	4.9	10	38.5	16	61.5	3.1 (0.3–30.8)			
Network size								**0.0024**		**0.0191**
Median (4) or less	244	45.7	60	24.6	184	75.4	1		1	
Greater than the median	290	54.3	107	36.9	183	63.1	**1.8 (1.2–2.6)**		**1.6 (1.1–2.4)**	
Received free condoms in the last 12 months								**0.0013**		**0.0254**
Yes	399	74.7	140	35.1	259	64.9	**2.2 (1.4–3.5)**		**1.8 (1.1–2.9)**	
No	135	25.3	27	20.0	108	80.0	1		1	
Received info on condom use and safe sex in the last 12 months								**0.0223**		
Yes	363	68.0	125	34.4	238	65.6	**1.6 (1.1–2.4)**			
No	171	32.0	42	24.6	129	75.4	1			
Ever spoken with a peer educator/outreach worker								**0.0040**		**0.0426**
Yes	363	68.0	128	35.3	235	64.7	**1.8 (1.2–2.8)**		**1.6 (1.02–2.5)**	
No	171	32.0	39	22.8	132	77.2	1		1	
Told by healthcare provider they had an STI in the last 12 months								**0.0004**		**0.0012**
Yes	75	14.0	37	49.3	38	50.7	**2.5 (1.5–4.1)**		**2.4 (1.4–4.1)**	
No	459	86.0	130	28.3	329	71.7	1		1	
Prefer to receive information about HIV from healthcare providers								0.4579		
Yes	429	80.3	131	30.5	298	69.5	0.8 (0.5–1.3)			
No	105	19.7	36	34.3	69	65.7	1			
Prefer to receive information about HIV from peer educators								**0.0267**		
Yes	311	58.2	109	35.0	202	65.0	**1.5 (1.1–2.2)**			
No	223	41.8	58	26.0	165	74.0	1			
Participated in transactional sex in the last 6 months^a^								**0.0010**		**0.0229**
Yes	47	9.0	25	53.2	22	46.8	**2.8 (1.5–5.1)**		**2.1 (1.1–4.1)**	
No	473	91.0	138	29.2	335	70.8	1		1	
Last sex with main male partner was CRAI^b^								0.0706		
Yes	90	17.9	35	38.9	55	61.1	1.5 (1.0–2.5)			
No	412	82.1	120	29.1	292	70.9	1			
Alcohol dependence^c^								0.9322		
Yes	98	18.4	31	31.6	67	68.4	1.0 (0.6–1.6)			
No	436	81.6	136	31.2	300	68.8	1			
Used non‐injection drugs in the past 6 months^d^								**0.0153**		**0.0042**
Yes	170	31.8	41	24.1	129	75.9	**0.6 (0.4–0.9)**		**0.5 (0.3–0.8)**	
No	364	68.2	126	34.6	238	65.4	1		1	
Major depressive disorder^e^								0.2984		
Major depressive disorder likely	83	15.5	30	36.1	53	63.9	1.3 (0.8–2.1)			
Not depressed	451	84.5	137	30.4	314	69.6	1			

Note: Statistically significant results are in boldface (*p* < 0.05).

Abbreviations: aOR, adjusted odds ratio; AUDIT, Alcohol Use Disorders Identification Test; CI, confidence interval; CRAI, condomless receptive anal intercourse; MSM, men who have sex with men; OR, odds ratio; PHQ‐2, Patient Health Questionnaire; PrEP, pre‐exposure prophylaxis; STI, sexually transmitted infection; TGW/GQ, transgender women/genderqueer.

^a^ *n* = 14 missing.

^b^ *n* = 32 missing.

^c^ AUDIT score ≥15.

^d^ Most reported non‐injection drugs used in the past 6 months included marijuana ([72.4%]; [123/170]) and tobacco ([62.4%]; [106/170]).

^e^ PHQ‐2 score ≥3.

^f^ Adjusted for all variables included in the final multiple logistic regression model.

### 3.5 PrEP awareness and use among PLHIV

In Harare, 12.0% of MSM (47/391) and 19.9% of TGW/GQ (50/251) who self‐reported an HIV negative or unknown status tested HIV positive. In Bulawayo, 12.4% of MSM (82/662) and 16.0% of TGW/GQ (8/50) who self‐reported an HIV negative or unknown HIV status tested HIV positive. Among the 187 PLHIV who self‐reported an HIV negative or unknown HIV status in both cities, 50.8% had heard of PrEP ([Harare: 72.2%; Bulawayo: 27.8%]; [MSM: 39.5%; TGW/GQ: 75.9%]); of these, 22.1% (21/95) had ever taken PrEP ([Harare: 24.3%; Bulawayo: 16.0%]; [MSM: 19.6%; TGW/GQ: 25.0%]). The main reasons for never starting PrEP among PLHIV included not knowing where to access PrEP (27.0% [20/74]), fearing side effects (27.0% [20/74]), not wanting to start PrEP (12.2% [9/74]), for other reasons (12.2% [9/74]) and not feeling at risk for HIV (10.8% [8/74]). Among PLHIV who reported ever taking PrEP and stopped (*n* = 13), reasons for stopping included experiencing side effects (8/13), not wanting others to know (3/13), no longer able to access PrEP (1/13) or for other reasons (1/13). Among PLHIV who were unaware of their status, had unsuppressed viral load and had reported ever taking PrEP, only a small number reported using PrEP in the past 6 months (6/14); of these, none reported taking PrEP the day of or proceeding the survey, though half reported taking PrEP within the last 2 weeks (3/6).

## 4 DISCUSSION

Our findings highlight gaps along the PrEP cascade for HIV‐negative MSM and TGW/GQ in our sample during early PrEP implementation in Zimbabwe. As HIV programs in Zimbabwe continue to expand PrEP services for KP, findings from this survey, including barriers to starting and continuing PrEP, can inform tailored interventions.

Where data are available, awareness of PrEP among MSM in sub‐Saharan Africa varies by country [21, 22], likely due to differences in country scale‐up and implementation of PrEP, country policies and PrEP communication strategies, and survey‐specific factors, underscoring the importance of country‐specific data to inform PrEP implementation. We found higher odds of PrEP awareness among participants with a larger network size, who had ever engaged with a peer educator, received free condoms or information on safe sex/condom use in the last 12 months or had ever tested for HIV, indicating those engaged in health services or more connected to other MSM or TGW/GQ were more likely to be aware of PrEP. This is consistent with PrEP implementation for these groups as rollout preceding the survey was limited to efforts aimed at demand creation at health facility‐ and KP organization‐level rather than through public awareness campaigns, and highlights the important role of peer educators in promoting PrEP for KP. While PrEP awareness was positively associated with exposure to HIV services, there were still substantial gaps in awareness among those who received HIV prevention information and those diagnosed with STIs in the past 12 months; these avenues provide opportunity to increase PrEP awareness and should continue to be prioritized in generating PrEP demand.

As seen elsewhere in sub‐Saharan Africa [21, 23, 24], willingness to take PrEP among our sample was high, though participants reported barriers to accessing and taking PrEP. Globally, common barriers to PrEP use include challenges accessing locations where PrEP is delivered, PrEP being used as evidence of sex work or other criminalized/illegal sexual activity and cost [25]. In this survey, key barriers to starting PrEP included not knowing where to access PrEP, concerns about side effects, low self‐perceived HIV risk and insufficient information. To increase awareness and uptake, demand creation messaging could be strengthened by providing information on PrEP accessible locations, PrEP eligibility and side effect mitigation approaches.

Our survey addresses the dearth of regional evidence on the PrEP cascade among TGW/GQ and highlights disparities in PrEP awareness and uptake between TGW/GQ and cisgender MSM as well as regional differences. Compared to TGW in South Africa, TGW/GQ in this sample had higher awareness and uptake of PrEP and greater willingness to take PrEP [26]. In our sample, TGW/GQ overall were more likely to have taken PrEP compared to their male counterparts and TGW/GQ in Harare and Bulawayo were more aware of PrEP than MSM in Bulawayo though awareness among TGW/GQ in both cities was comparable to that of MSM in Harare; this may be attributable to the larger KP program in Harare where funding and targets are greater than those in Bulawayo. A “one size fits all” approach to demand creation messaging is insufficient and our findings emphasize the need for tailored interventions to promote PrEP among KP; these may include advertisements through social media applications, dating applications and other online platforms considering sensitivities in this context, including criminalization of sex between men, homophobia and transphobia, and little to no legal protections for these groups [4, 5].

In our sample, PrEP uptake was higher among participants reporting recent (≤6 months) transactional sex or recent (≤12 months) history of an STI diagnosis. The relationship between transactional sex or STIs and PrEP uptake cannot be causally assessed in this survey, but there is no evidence of behavioural risk compensation elsewhere [27], suggesting that Zimbabwe may have been more successful in reaching HIV‐negative MSM and TGW/GQ at greatest risk of HIV acquisition with PrEP than those reporting fewer sexual risk behaviours. Greater uptake among sex workers may be a result of Zimbabwe's robust HIV program for sex workers [28]. Moreover, these data may provide evidence of fidelity of PrEP eligibility screening in survey sites [8] and suggest that concomitant STI‐to‐PrEP interventions may enable PrEP uptake. Further study is warranted to understand potential risk compensation in this context.

Despite promising evidence in reaching HIV‐negative MSM and TGW/GQ most at risk for HIV with PrEP, half of participants who were newly diagnosed with HIV during the survey—those who could have most benefitted from PrEP—were unaware of PrEP. Nearly, one‐quarter of those newly diagnosed and aware of PrEP had taken PrEP; of these, six reported taking PrEP in the past 6 months and three in the last 2 weeks. In our sample, reasons for never starting PrEP were similar for both HIV‐negative participants and PLHIV. Though no participants reported stopping PrEP due to seroconversion, recent (≤6 months) PrEP use among few PLHIV underscores the need for more thorough clinical follow‐up and monitoring of PrEP, including repeat HIV testing, assessment of acute HIV infection and support for PrEP adherence. With more than half of PLHIV who reported ever using PrEP ceasing use due to side effects, counselling on side effects upon PrEP initiation and side effect mitigation techniques, such as use of over‐the‐counter medications for symptoms, could be strengthened to support PrEP retention [29].

This survey has limitations. RDS relies on assumptions that the target population is networked and that those recruited through RDS can accurately report their network size. Our sample did not reach convergence on key estimates related to primary study objectives, indicating that our sample may not be representative of the broader target population as the sample was influenced by the purposively selected seeds. Due to small numbers, we were unable to run models separately by KP or for those currently on PrEP. For this analysis, “eligible” for PrEP was defined as participants with an HIV‐negative result as participants were not screened for signs of acute HIV infection or creatinine clearance. Participants were not explicitly asked whether PrEP was offered to them by a provider, which could provide an additional insight into PrEP availability. As described earlier, the cross‐sectional study design inhibits causal interpretations and behavioural risk compensation for PrEP users is unknown in this context. Moreover, data were self‐reported and may be subject to social desirability bias. Despite limitations, this large RDS study provides evidence to assess and guide PrEP scale‐up among MSM and TGW/GQ in Zimbabwe, populations disproportionately affected by HIV and underrepresented in HIV research.

## 5 CONCLUSIONS

This survey identified gaps in PrEP awareness and uptake among and between MSM and TGW/GQ in this sample as well as key barriers to starting and continuing PrEP, including not knowing where to access PrEP, side effects, low‐risk perception and insufficient information. Overall, participants engaged in health services, including engagement with a peer educator, or more connected to other MSM or TGW/GQ were more likely to be aware of or had ever taken PrEP, underscoring the important role of peer educators to address gaps across the PrEP cascade. Findings on regional and KP‐specific disparities emphasize the need for tailored interventions to promote PrEP. As Zimbabwe continues to expand PrEP services, results can inform interventions to increase PrEP awareness and uptake among KP.

## COMPETING INTERESTS

The authors declare no competing interests.

## AUTHORS’ CONTRIBUTIONS

LEP, TGH, IC, MM, OM, JHR, CS, GM and AJH conceptualized the survey. IC, MM and PG managed data collection. LEP conducted data cleaning and analysis. YW provided additional analytic support. LEP and AJH drafted the manuscript. All authors critically reviewed and approved the final version of the manuscript.

## FUNDING

This project was supported by the U.S. President's Emergency Plan for AIDS Relief through the CDC under the cooperative agreement U2GGH001939.

## DISCLAIMER

The findings and conclusions in this manuscript are those of the authors and do not necessarily represent the official position of the funding agencies.

## AUTHOR INFORMATION

The protocol was also reviewed in accordance with the U.S. Centers for Disease Control and Prevention (CDC) human research protection procedures and was determined to be research, but CDC investigators did not interact with human subjects or have access to identifiable data or specimens for research purposes.

## Data Availability

Data are available upon request. Requests should be sent for review to Dr. Brian Moyo (Epidemiologist, AIDS and TB Programme at MOHCC) at boyobk1@gmail.com. Individuals seeking access to data will need appropriate IRB and institutional (ICAP at Columbia University, MOHCC, CDC) leadership approval, which will be facilitated by Dr. Brian Moyo.
